# Theory-driven formative research to inform the design of a national sanitation campaign in Tanzania

**DOI:** 10.1371/journal.pone.0221445

**Published:** 2019-08-23

**Authors:** Alexandra Czerniewska, Winnie C. Muangi, Robert Aunger, Khalid Massa, Val Curtis

**Affiliations:** 1 Department for Disease Control, London School of Hygiene & Tropical Medicine, London, England, United Kingdom; 2 Department of Economics, University of Dar es Salaam, Dar es Salaam, Tanzania; 3 Environmental Health and Sanitation Section, Ministry of Health, Community Development, Gender, Elderly and Children, Dar es Salaam, Tanzania; University of Texas Medical Branch at Galveston, UNITED STATES

## Abstract

**Introduction:**

There are gaps in global understanding about how to design and implement interventions to improve sanitation. This formative study provided insights for the subsequent redesign of a government-led national sanitation campaign targeting rural populations in Tanzania.

**Methods:**

The Behaviour Centred Design approach was used to investigate the determinants of toilet building, improvement and use. Varied, novel, and interactive research tools were employed in fifty-five households in two regions of rural Tanzania. Results were analysed to articulate a Theory of Change, which then informed intervention design.

**Results:**

Participants valued hard work, enterprise, and improving their lives over many years. They wanted better toilets but felt no urgency to act quickly. A common emotional motivator for improving toilets was to protect children from disease (Nurture) but this was insufficient to drive rapid change. Disgust with traditional toilets meant they were built at a distance from the house: an ‘out of sight, out of mind’ attitude. Other powerful motives included the desire to improve living conditions (Create), and to become a modern Tanzanian (Status), albeit without ‘showing off’. Construction costs and water scarcity were the main stated barriers. Receiving information about realistic costs, support accessing materials, and visiting better latrines elsewhere were commonly reported reasons for improving latrines.

**Conclusions:**

The resulting Theory of Change recommended that the intervention should surprise people with a novel conversation about toilets, promote toilets as a means of conferring status, and introduce a perceived urgency to ‘act now’. It should suggest that modest improvements would lead to a better life. Feelings of disgust and fear with poor quality toilets should be amplified, and barriers lessened through promoting transformational toilet improvements, and improving access to modern toilet products. This research provided considerable insight into sanitation behaviours in rural Tanzania, which informed creative intervention design.

## Introduction

Sanitation remains a major global challenge. Almost a quarter of the world’s population lack access to a facility that hygienically separates excreta from human contact [1]. In sub-Saharan Africa over half of the population lack access to an ‘improved’ sanitation facility at home (see Box 1 for definitions) [1]. Inadequate sanitation causes an estimated 280,000 deaths from diarrhoea every year [2], mostly in low-income countries. Interventions that move people from open defecation or ‘unimproved’ sanitation facilities to ‘improved’ facilities could reduce diarrhoea risk by about a quarter [3].

**Box 1. Sanitation definitions [22]**

**‘Improved’** sanitation facilities are defined as those designed to hygienically separate excreta from human contact. Examples include: flush/pour, flush to piped sewer system, septic tanks or pit latrines; ventilated improved pit latrines; composting toilets, or pit latrines with cleanable slabs for squatting and covering the pit. In Tanzania, such latrines are labelled ***‘choo bora’*** (good toilet).

**‘Unimproved’** facilities include latrines without slabs, hanging latrines or bucket latrines. In Tanzania, these latrines are called ***‘choo asili’*** (natural/traditional toilet). These are usually simple pits covered with wood and earth, often with superstructures made from makeshift materials.

Poor sanitation is also linked to soil-transmitted helminth infections [4, 5], neglected tropical diseases [6], malnutrition, and stunting [7, 8], although these findings are not consistent across all contexts [9, 10]. Improving sanitation can have a disproportionately positive effect on women and girls by enabling better menstrual hygiene management [11, 12], reducing stress [13], or by challenging traditional gender roles and inequalities [14, 15].

National sanitation programmes usually attempt to encourage householders to improve their toilets (often alongside other waiter and hygiene improvements)through a mix of individual and communal persuasion, subsidy, demonstration and enforcement [16, 17]. Whilst an approach to ‘triggering’ communities to take action known as Community-Led Total Sanitation (CLTS) has been applied worldwide over the past decades [18–20], we hypothesise that new approaches based on behavioural science are needed to achieve large scale, sustained changes in latrine quality and use. This requires systematically searching for insights into potential drivers of behaviour change, and carefully constructing intervention ‘theories of change’ [21] reflecting global insights and local contexts.

### Study objectives

This formative research study aimed to provide the insight needed to design new creative material for the National Sanitation Campaign in rural Tanzania. We describe how the results of this study informed a theory of change that was used by the Tanzanian Government (in partnership with commissioned partners) to create a high profile, multi-channel campaign including mass media and associated community mobilisation activities.

### Tanzania

Tanzania is making rapid progress on many human development and economic indicators [23] but sanitation remains an issue. The *Mtu ni Afya* (*Man is Health*) campaign in the 1970s and the first phase of the *National Sanitation Campaign (NSC)* from 2011 to 2016 achieved high latrine coverage relative to other sub-Saharan countries. *Mtu ni Afya* achieved this through radio, listener groups, and other channels that aimed to change behaviour though empowerment and peer pressure, rather than through information about germs or use of hardware subsidies. The first phase of NCS used a combination of CLTS, social marketing and behaviour change communication [24].

However, many toilets were still of poor quality, potentially conveying little health benefit in comparison with open defecation (Wolf et al., 2018). In 2017 only 21% of people in rural Tanzania had access to good quality toilets [1], with 16% practicing open defecation. According to a 2014 evaluation, about half of rural latrines were reported to smell (48%) or have flies (52%), and nearly a quarter had visible faeces outside the cubicle (24%) [24].

### Theoretical foundations: Behaviour Centred Design

We used the Behaviour Centred Design (BCD) approach [25] which has previously been employed to alter a range of public health behaviours [26–29]. BCD employs five design steps to **A**ssess, **B**uild, **C**reate, **D**eliver and **E**valuate a behaviour change program [25].

Behaviour is determined by a set of physical, biological, social and psychological factors [30–34]. BCD recognises that most behaviour takes place in ‘behaviour settings’, which are “small-scale social systems composed of people interacting with one another and with inanimate objects to carry out a regularly occurring, prescribed behavioural sequence, or program, within specifiable time and place boundaries” [35]. BCD also recognises fifteen universal human motives [36] including Lust, Comfort, Fear, Disgust, Love, Nurture, Affiliation, Hoard, Create and Justice.

Behaviour settings and motives can be explored and described in formative research (FR) through a simple set of parameters: physical (stage, props, infrastructure), biological (competencies), psycho-social (roles, norms, scripts), and behavioural (routines) [37, 38]. BCD FR (the Build step) is generally qualitative, in-depth and observational, preferring methods that capture insight about actual behaviour in its setting over those that employ standardised self-report and statistical comparisons (e.g. [31, 39, 40]). It employs a wide range of tools to actively engage participants around their routines, motives and environmental conditions, going beyond the focus groups and in-depth interviews which are standard in most FR (e.g.[41–43]). The process does not assume that there is only one way to change behaviour, but seeks insights concerning powerful, plausible and feasible routes to behaviour change.

Before conducting this study we completed a rapid review of relevant published and grey literature and a two-day ‘framing’ workshop (the Assess step) with stakeholders in Tanzania to agree a precise set of target behaviours, identify knowledge gaps, and develop hypotheses for further exploration.

**Fig 1:**
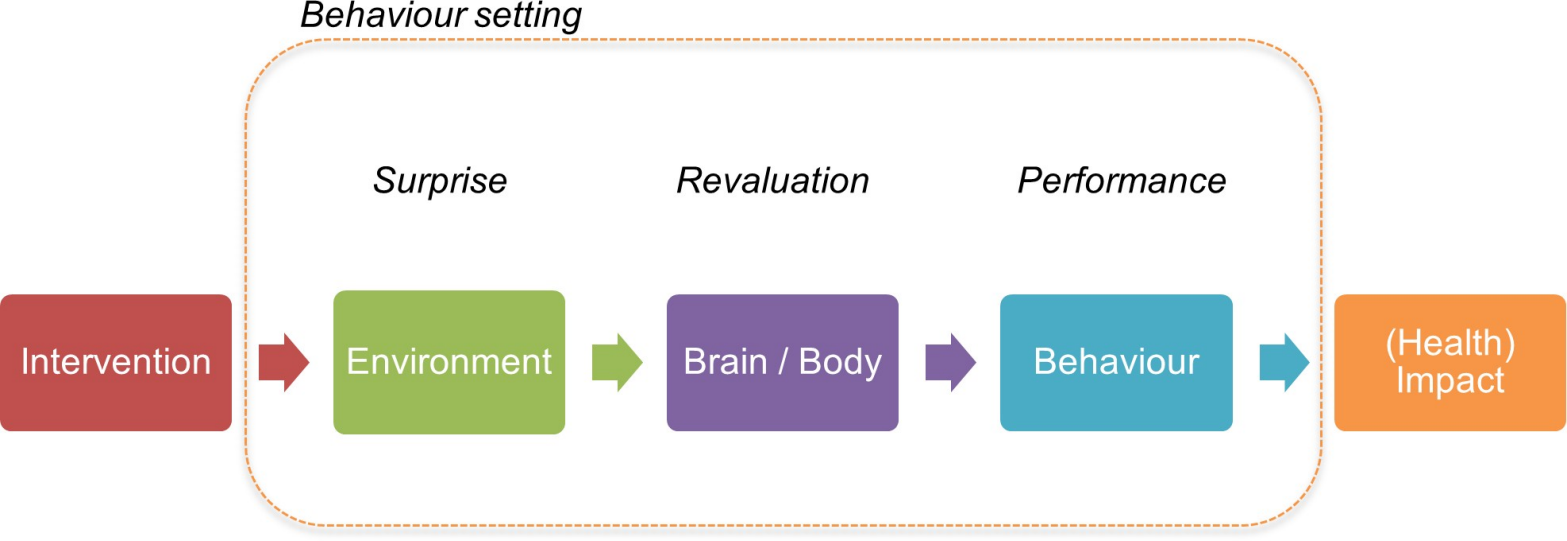
Generic BCD Theory of Change.

FR findings are coded using a checklist from which a theory of change is created to guide the creative process. We start from the generic BCD theory of change which proposes that improvements in health outcomes should occur if the intervention can change the immediate environment (physical, biological, and/or social) of the target audience in a perceptible and salient way (BCD calls this a ‘*surprising’* intervention [25]). Thus gaining attention and providing new stimuli, the intervention causes the target audience to *revalue* behaviours due to a psychological and/or a physical change (their motivations or capabilities). Surprise and revaluation leads to sustained *performance* of the new behaviour (Fig 1). BCD also incorporates a design process for creating novel and disruptive interventions from FR findings.

## Methods

### Study setting and sampling

We conducted formative research in August 2017 in two regions of Tanzania: Morogoro (Mvomero and Morogoro districts) and Mwanza (Ilemela and Misungwi). We purposively selected four districts, and subsequently seven rural and three peri-urban villages differing in remoteness, sanitation coverage and exposure to previous sanitation promotion. To select households, each team walked in a different direction (transect) from the starting point, accompanied by a local volunteer who we asked to take us to the first household with children under five on the transect. After conducting an interview, each team repeated this process. Our sampling strategy was to generate insights from people representing our ‘typical’ campaign targets for the creative process, not to represent the full variety of the Tanzanian population.

### Recruitment and consent

We interviewed one adult per household. We explained the study in the local language and obtained informed, witnessed, written consent. We received ethical permission from the London School of Hygiene and Tropical Medicine (13810) and the National Institute for Medical Research, Tanzania (NIMR/HQ/R.8a/Vol.IX/2546). We also received permission from the Ministry of Health, Community Development, Gender, Elderly and Children, the Prime Minister’s Office for Regional Administration and Local Government, regional and district officials, and village leaders.

### Study tools and team

We employed varied, novel, and interactive tools in a flexible and iterative protocol derived from consumer research called ‘sequential recycling’ [44]. We adapted or discarded some tools once they were no longer producing new insights, and we added others as new themes and questions emerged. We used between four and eleven tools per participant.

Table 1 gives a brief overview of the purposes, methods and sample for each tool. More details of the tools employed in BCD formative research can be found at www.lshtm.ac.uk/bcd. Each tool had a specific purpose in understanding the ‘behaviour setting’ around the behaviours of toilet construction and use. Where we identified other behaviours that were inextricably linked to the same behaviour setting (e.g. handwashing) these are reported.

**Table 1:** Tools used in household (HH) interactions.

TOOL	PURPOSE	METHOD	No. HHs
**Toilet History**	Understand the history of current and previous latrines in a household plot.	Participants describe their experience with toilets on the plot; including how and when existing toilets were procured.	53
**Daily Routine/ Scripting**	Elicit the normal sequence of events in everyday life, with emphasis on target behaviours.	Participants list and discuss the activities they did yesterday, and then consider where sanitation behaviours fit into this routine.	50
**Household observation**	Record salient physical features of the setting.	The toilet is visited and photographed. Its characteristics are noted including structure, materials, quality, and availability of water or handwashing facilities.	43
**Finances/ Decision Making**	Understand how households save and spend, particularly with reference to sanitation.	Participants discuss their methods of saving and how the household makes spending decisions.	41
**Toilet Costing**	Understand perceived cost of improving latrine and knowledge of latrine elements.	Participants compare pictures of toilet components and estimate relative and absolute costs for each.	32
**Touch Points**	Map channels of communication with target audiences.	Sources of relevant information and social influence, including events, media, community groups, savings and government outreach are documented.	29
**Superpowers Game**	Elicit relative importance of motives.	Participants bid sums of (play) money to obtain ‘powers’ represented by the motives e.g. never being hungry; being respected by peers	25
**Toilet Improvement Ranking / Build your own Toilet**	Understand the relative importance of types of toilet improvements.	Participants are shown pictures of potential improvements to toilets to discuss and rank by importance.	25
**Windfall Exercise**	Determine the value of toilet investment in comparison with other options.	Participants are given increasing sums of (play) money and asked how they would spend it.	24
**Three Wishes**	Understand what aspirations parents have for their children.	Participants describe their top three aspirations their children’s futures.	17
**Sanitation Motive Mapping**	Elicit relative motivations for building or upgrading a *choo bora*.	Participants are shown an cartoons of toilet building with outcomes showing potential benefits/rewards according to the motives set, to discuss and rank.	11
**Norms**	Understand expectations on beliefs, sanctions and moral status of a behaviour.	Participants are asked to estimate what proportion of people in their village carry out the target behaviours. Questions based on Bicchieri, Lindemans [45].	16
**Toilet personification**	Understand social implications of toilet choices.	Participants are shown images of different toilets and describe the person that would choose and own them.	10

The research group consisted of three teams of two people: a Tanzanian social scientist who spoke the local language and an international researcher with experience of behaviour change (Indian and UK nationals.

### Data analysis

Research teams took notes by hand (in English) during the interviews and typed them up daily. Analysis took place in two phases. A rapid debrief meeting was held within a week of returning from the field, where participants discussed their most salient findings and these were organised into a preliminary checklist. In a second phase of more academic analysis we used Nvivo 11 software to code and organise the data according to the BCD Checklist headings (see Table 2) and analysed this data thematically.

**Table 2:** Findings organised by the BCD checklist.

Component	Sub component	Findings
**Behaviour Setting: physical components**	**Stage:** the physical ‘backdrop’ to performing behaviours	• Houses were usually constructed from higher quality materials than the toilets. • Latrines were located at a substantial distance from the home to avoid smell and flies. • Space was not a major constraint in rural areas, but compounds were often full of holes where latrines had collapsed or filled up.
	**Infrastructure:** physical structures necessary to perform behaviours	• Latrine quality varied: flooring was often earth tamped over a wooden structure, and walls were made of wood, mud, or cloth. Improvements such as roofs, toilet covers, or cleanable flooring were rare. • There was rarely a place for handwashing around the latrine. • A lockable door and a cleanable surface were the most highly valued components of an improved latrine. A ceramic pan, u-bend, and offset pit were least valued. • Seated pans were considered inappropriate for outdoor latrines. • Water was scarce, hampering the ability to flush and clean toilets. • Around 1/3 of total household water was reserved for latrine use including handwashing (20 litres for a pit latrine and up to 60 litres for a pour-flush latrine per day).
	**Props:** objects necessary to perform behaviours	• Small containers were used to bring water for anal cleansing. • Menstrual materials were either reused or disposed of in the latrine. • Pit latrines were commonly swept with local brooms, sometimes using water, powdered soap or detergent. Improved latrines were cleaned with water and powdered soap and brushes or rubber scrapers.
**Behaviour Setting: behavioural components**	**Routine:** the sequence of behaviours regularly performed in a setting	• Household latrines were most commonly used early in the morning, after waking, or after completing morning chores (collecting water, cleaning house).
**Behaviour Setting: psychological components**	**Roles:** the part each person is expected to play with regards to the behaviour setting.	• Men took the decision to build or upgrade the latrine, but women could apply pressure to do so. • Pit latrines were usually constructed by men from the household from local materials, whereas a mason was needed to construct an improved latrine. • Women generally performed the day-to-day roles of water collection, disposing of child faeces and cleaning the latrine. • Children sometimes brought water to the latrines for adults.
	**Norms:** the informal rules governing actions taken (and not taken) in the behaviour settings	• Open defecation was rare and generally frowned upon. • There were different norms around toilet quality within communities. Poor quality toilets were seen as normal and ‘good enough’ for poor people, although bad smells would invite social judgement. Wealthy people were respected and expected to have better quality latrines. • Latrines could be used by passers-by or neighbours.
	**Executive brain:** explicitly represented mental knowledge, beliefs and planning	• Money was invested in income- generating activity and home improvement before latrines. • Participants were aware that they could be fined for not having a latrine. • There was poor knowledge of the cost of constructing an improved latrine, which was often perceived to be prohibitively high. • Participants considered the amount/cost of water needed in their choice of latrine design but not the costs of emptying pits. • Participants committed to a design, a budget and a mason for an improved latrine upfront, but could collect materials for construction slowly over time.
	**Motivated Brain:** the goal or benefit an individual will gain from performing a behaviour (e.g., emotional drivers, intrinsic rewards).	• Key motives for building an improved latrine were to protect children and family (Nurture), durability (Hoard), better experience (Comfort; Disgust, Fear), disease prevention (Disgust) and an improved living environment (Create). • Women were motivated by Nurture more than men (the decision makers) who were more commonly motivated to improve living conditions, learn skills and buy mobile phones or modern furniture (Create, Play and Hoard). • Improved toilets could confer respect (Status). However, these motives were mitigated by social norms and negative social judgement if you were perceived to be showing off (seeking Status) or wrongly prioritising income. • Social motives (Affiliation, Attract, Justice, Love) were less cited than personal ones
**Biological (body)**	**Competencies:** the skills required to perform a behaviour	• Most males in households had the knowledge and skills to dig and construct basic pit latrines as they had often contributed to building their own homes. • There was an incomplete understanding of the component parts and costs needed for constructing an improved latrine (this required the skills of a mason). • Receiving information on design and cost, and CLTS triggering were reported reasons for building a latrine. • Training and promotional materials to improve knowledge of improved latrines were rarely available.
**Context (outside of the setting)**	Physical environment	• Life in rural Tanzania is improving, houses are being upgraded and ownership of goods increasing. A lot of building work was in evidence • Scarcity of water is a barrier to upgrading to an improved latrine, which takes more water to flush and clean
	Biological environment	• The environment around homes is contaminated with human and animal faeces.
	Social environment	• Neighbours came together at times of sickness and death, for agriculture, or to manage community schools, but not for latrine building. • Loans were not considered for latrines because interest rates are high (saving was preferred). • Recall of visits by local health officers was low, but respondents reported regular attendance at village meetings and church, where sanitation and hygiene issues were occasionally discussed. • Discomfort with talking about defecation and latrines was evident.

## Results

### Sample characteristics

The research team conducted interviews with 29 participants in Morogoro region and 26 in Mwanza region (33 women and 22 men in total). Participants were aged between 20 and 69 years. Three quarters of the participants were farmers, but other occupations included a shop owner, mobile phone agent, and tailor. Most (71%) participants had completed primary education only, 7 had completed secondary education, and 6 had neither. Most participants were either Christian (62%) or Muslim (35%). About half of participants were of Sukuma ethnicity (from Mwanza), 14 were Luguru (from Morogoro) and the remaining 15 were from 10 other ethnic groups. Three quarters of participants lived with a partner. The modal number of children in each household was three, and the maximum was 10.

We observed unimproved pit latrines in 31 households (56%), improved latrines in 22 households (40%), and no latrine in the remaining 2 households (4%). The mean monthly household income was 200,000 Tanzanian Shillings (US$89 at October 2017), with a range from 0 to 1,000,000 Tanzanian Shillings (US$0–445 US) per month, and was reported to be highly seasonal. We collected data on asset ownership to give an idea of relative wealth, and found that most households owned mobile phones and radios, but few owned motorbikes, TV or fridges. Solar power was commonly used for lighting. Women tended to describe their daily routines as centring on the home (cooking, cleaning, caring for children), but with regular trips to water collection points, food markets, or to work in small businesses. Male participants were more likely to spend their days working away from home (mostly farming) and were more likely to report socializing in the evenings.

We summarise our results in Table 2, organised according to the categories of the BCD checklist.

### Behaviour settings: Physical components

#### Stage

Latrines were usually located at some distance from the main dwelling. According to one participant: *The latrine is not built close to the house because toilets usually stink*, *hence it is best that it is built far from the house* (Household number 30; HH30). The main house was usually the highest quality structure on the stage, often made of concrete blocks with a galvanised steel roof and we saw evidence of lots of recent home improvement. Meanwhile, latrines were generally constructed from lower quality materials– e.g. wood, mud, and stone gathered at no cost from the surroundings. Latrine pits were reported to be prone to collapse, especially in the rainy season, leaving multiple rubble filled holes in the compound.

#### Infrastructure

The type and quality of latrine infrastructure varied. Basic pits latrines were typically shallow and unlined. Wood planks covered with tamped earth tended to be used to cover the pits, which were vulnerable to insect, fungal and rain damage and were hard to keep clean. The superstructure was often constructed of branches or tarpaulin, sometimes with no roof or door. Better quality latrines observed in the households visited typically had lined or unlined pits more than eight feet deep, sometimes with offset pits and ventilator pipes. These latrines had simple concrete floors, with ceramic pans and block walls, but often had a flimsy door without a lock, and sometimes no roof. We saw no examples of other latrine types e.g. composting toilets or urine-diverting dry toilets.

We rarely observed any improvements or ‘beautifying’ features to toilet infrastructure. Of the 31 households with a basic pit latrine, only four had any features of an improved latrine, e.g. robust walls, lockable doors or cleanable floors. Only two households had a toilet cover to put over the hole when not in use. There was rarely a designated space for handwashing such as a fixed sink or handwashing stand. We interviewed one disabled participant, who was unable to walk and found a basic pit latrine dangerous and difficult to use. His brothers had come from the city with a craftsman and constructed an improved latrine for him, but we observed no features designed to make it more accessible.

Participants reported (through the ‘Toilet Improvement Ranking/Build Your Own Toilet activities) that the most valued latrine improvements were a lock (on the outside of the door to keep out passers-by who made the latrine dirty and filled it up), and a cleanable ground surface. Other desired features were a vent pipe, a window, and a covered pit to reduce visible dirt and smell. Participants placed a lower value on having a ceramic pan, u-bend, or offset pit (i.e. features of a pour-flush system), due to problems with water scarcity - *I like the pan*, *but there is a problem*, *can I access water easily*? *(HH25)*. Only two participants saw a pedestal seat as an improvement over a squatting slab– one for mobility issues, and one who told us that *the world is changing fast*, *hence it is best to keep up with modernization (HH2)*. For many households the ideal toilet was part of a bathroom block with both a toilet and shower cubicle.

#### Props

Props for latrine use included water and small containers for anal cleansing and handwashing, sandals reserved for toilet use, and an additional cloth to cover the doorway for privacy. Some participants described sprinkling ash, salt or detergent into the pit latrine after use to avoid bad smells.

Pit latrines were commonly cleaned by sweeping with locally made brooms, and sometimes with water, powdered soap or detergent. Improved latrines were cleaned with water and powdered soap and brushes or rubber scrapers. Participants reported using around one third of their household water supply for the latrines – 20 litres per day for a pit latrine and up to 60 litres for a pour-flush latrine.

### Behaviour settings: Behavioural components

#### Routines

The most frequently reported times for toilet use were directly upon waking, or after completing morning chores (e.g. collecting water, cleaning house). All household members reported using household latrines, except young children who usually defecated on the ground in the compound.

There were no routines around maintenance and repair of latrines. Few participants were aware of any latrine emptying services, or considered them necessary. If a basic pit latrine filled up, it was considered easy to dig a new one and cover the full pit. Respondents had not thought about whether an improved latrine would fill up, nor what they would do in such a case.

### Behaviour settings: Psychological components

#### Roles

Men generally held the financial decision-making power to build or upgrade a latrine, although women could apply pressure on men to do so. Males in the household usually designed and constructed the latrines (or oversaw design and construction if a mason was employed). Women generally performed all of the day-to-day roles in the behaviour setting, including collecting and managing water in the household and cleaning the latrine. Mothers were usually responsible for disposing of child faeces into the latrine, burying or throwing them into the bush: *When children use potty and when women don’t clean it is unhealthy and bad*. *It is better for children to go outside and then for women to throw it in the toilet (HH5)*. Children were sometimes called to bring water and/or soap for parents and other adult household members to hand wash after using the toilet.

#### Norms

Open defecation was perceived to be rare, and was generally frowned upon. Of eleven participants asked, nine thought one ‘*should*’ have an improved latrine, but five said it was *‘OK’* to have a basic pit latrine if you could not afford to upgrade. There were different accepted norms for different members of the community: wealthier people were expected to have better quality latrines and were respected for this.

Participants who did not agree it was ‘OK’ to have a poor quality latrine said it was *not good for the surroundings and hygiene (HH44)* and that *what you leave behind is waste and can affect other people (HH46)*. Bad smells emitting from a latrine would attract negative social judgement. Some participants approved of financial sanctions for open defecation or if a poor quality latrine affected others. However, fines to encourage people to upgrade from basic to improved latrines were thought to be overly dictatorial, and likely to be ineffective: *If people are fined it wouldn’t make them build a* choo bora. *It’s a personal choice so people shouldn’t interfere (HH44)*.

Most households accepted that passers-by and neighbours would use the latrines: *For the time being I am using neighbour’s choo asili*. *It is normal to help each other in this village (HH41)*. However, this was occasionally a source of tension: *Right now I’m using toilet of neighbours*, *down by the road*, *they put up with it*, *but they don’t like it (HH24)*.

#### Executive brain: Beliefs and plans

Around half of participants were actively planning to improve their latrine. The main reported reason was to avoid fines from village authorities. Participants also said that it was important to avoid diseases, so that they could have long lives and look after their families.

There was huge variation in knowledge and belief about the cost of building an improved latrine. Those who had no toilet said one would cost from 80,000 to 1,000,000 TZS (US$36445), whilst reported actual costs (by those who had an improved latrine) varied even further – from US$58– 713. Many participants said that construction costs were prohibitively high. Participants included the costs of increased water consumption associated with an improved latrine, but did not mention any costs associated with eventual emptying of the pit or rebuilding a toilet that had filled up.

Savings and surplus income (after meeting basic needs) tended to be invested towards income- generating activities (animals or agricultural land) and home improvement rather than in in latrine improvement. In the ‘Windfall’ exercise, participants did not choose to spend their hypothetical money on their latrine until the sum offered was large (nearly US$200). Loans were not considered as a source of funds to improve latrines, due to the high interest rates charged by microfinance lenders. Given the seasonal nature of their income, most people collected materials slowly over months or years, in the same way that they collected materials to build houses. More planning was needed for an improved latrine because the household would commit to a design and budget and contract a mason from the outset.

#### Motivated brain

We captured motives related to toilets through Motivation Mapping tools, the ‘Toilet Personification’ tool, and the ‘SuperPowers’ game (Table 3), with further insights revealed through thematic analysis of interview scripts.

**Table 3:** Superpowers game results.

Motive	Number (%) of bills spent (n=25; total bills =250)	
**Nurture: to ensure my children will always be happy, safe and successful **	40	(16)
**Disgust: to never catch a disease from anyone **	38	(15)
**Create: to always be able to create a good physical environment to live in **	30	(12)
**Play: to always be able to learn new skills easily **	29	(12)
**Hoard: to always have all the stuff I need to be prepared for any situation**	25	(10)
**Hunger: to never feel hungry or thirsty again **	19	(8)
**Fear: to always be safe from attacks or accident**	15	(6)
**Curiosity: to always be well-informed about what’s going on in the world **	10	(4)
**Status: to always be esteemed and respected by others **	11	(4)
**Comfort: to never feel physical discomfort **	8	(3)
**Justice: to make others always be honest and fair **	6	(2)
**Affiliation: to make others like me and want me in their group **	6	(2)
**Attract: to always be beautiful; able to attract the same/opposite sex **	5	(2)
**Love: to always be loved by the (wo)man of my dreams **	3	(1)
**Lust: to always have an active sex life **	0	(0)
**Bills not spent**	5	(2)

Participants (particularly female participants) most desired the ‘superpower’ to provide education, food and protection for their children, and to make them happy (Nurture motive). This was also a key reason for choosing an improved latrine. Participants also feared children falling in to pit latrines and feared falling ill from infection. Other motives for improving latrines included better durability of the latrine (Hoard motive), to provide a better user experience; avoiding unpleasant smells and sights (Comfort and Disgust motives), to prevent spread of disease (Disgust motive).

Participants also desired the ‘superpower’ to improve their lives by starting economic ventures in farming and retail (Create motive). Male participants particularly valued the ability to make improvements to their surroundings, and to learn new skills that would enable them to generate more income, and therefore obtain items such as mobile phones, televisions and furniture associated with more modern, urban lifestyles (Play and Hoard motives).

Gaining respect and avoiding shame (Affiliation and Status) were sometimes important reasons for building an improved latrine. Having a presentable toilet could reduce shame in front of guests and visitors and could improve the way they were perceived by others: *They will think I am protecting the environment (HH39); People will think she is rich; she has money (HH45)*. However, many participants interpreted the Status motive as being about ‘showing off’ to others, something that was frowned upon: S*howing off is disapproved of (HH37)*. *Showing off is not a good tradition for us*. *If you want people to like you*, *you have to teach them*. *Even if you have a beautiful house*, *you should not tell people the cost*, *you should just show them how you did it*, *and where to find the mason (HH54)*. Participants also worried that neighbours would think they were misusing their money: *If I spent 1million shillings on a new toilet*, *people will think that my business is progressing and that I am better off*. *But if I spent that on the* choo bora *now*, *while my house is incomplete*, *people would not understand*. *They would think my priorities were wrong (HH36)*.

### Behaviour setting: Body (competencies)

Most adult men could dig and construct basic pit latrines, but digging and lining a pit for an improved latrine required the skill of a mason. Participants were familiar with most of the elements of an improved latrine, but had a better understanding of straight-to-pit technologies than of offset pits. Knowledge about toilet design came piecemeal from government meetings and discussions with local government officials, neighbours and masons. No participant reported receiving comprehensive information or relevant training. Some participants cited receiving this information as the reason they decided to construct or improve their latrine. Some said that they had been promised training by government officials, but as this had not materialized, they had decided to put their toilet improvement plans on hold. Everyone was expected to be able to use an improved latrine, except for very young children, and one participant described helping a disabled adult family member.

### Context

#### Physical

Participants cited water scarcity in dry seasons as a barrier to owning an improved latrine. Access to local water supplies was reportedly better in Morogoro region than in Mwanza region where households routinely reported travelling for thirty minutes or more to the nearest water source, making multiple trips of up to three hours each. On the other hand, we saw a lot of building work in evidence in these rural areas as people upgraded the quality of their houses, and ownership of goods was reported to be increasing.

#### Biological

Due to the lack of hygienic sanitation and rubbish collection facilities, there was organic waste present in many locations. Animals such as poultry, dogs, goats and sheep were kept in some compounds, causing faecal contamination.

#### Social

Few participants could recall being visited by local health officers. Participants reported regular attendance at village meetings and church, where sanitation and hygiene issues were occasionally discussed. Many women were members of saving and loans (*vikoba*) groups, but the loans available were too small or short-term to consider using for improving latrines.

Neighbours reportedly came together to help each other at times of sickness and death, to clear land for agriculture, and to raise funds for school buildings. All participants that we asked agreed with the statement that ‘around here, people take care of each other when they are in need’: *Especially when sick*, *or having funeral (HH45); digging a grave or bringing food when someone dies (HH43)*. However, neighbours rarely discussed latrines and participants were uncertain as to how they could ‘come together’ to build toilets. In general, there was a cultural discomfort with talking about faeces and latrines.

## Discussion

We designed this study to systematically generate insights for the second phase of Tanzania’s National Sanitation Campaign The first task of a behaviour change program is to define the target behaviour with sufficient precision to be measurable [21, 46]. Here, the target behaviour was to improve a household’s toilet to meet international standards of ‘improved’ [22], or to get one in the first place, if they did not already have one.

The second task is to disrupt the usual behaviour setting. Following BCD’s generic theory of change (Fig 1), our task was to create *surprising* interventions that could drive changes in the wider environment and the specific toilet-related settings of target audiences such that the target behaviour became more highly *valued* and could be *performed* more easily (surprise, revaluation and performance being the three features of a successful behaviour change intervention described by the BCD approach [25]). Our brief was that the interventions also had to be appropriate for a government-led, national-level campaign, which could be delivered at scale utilising mass media, and with no provision of subsidy for households.

**Fig 2:**
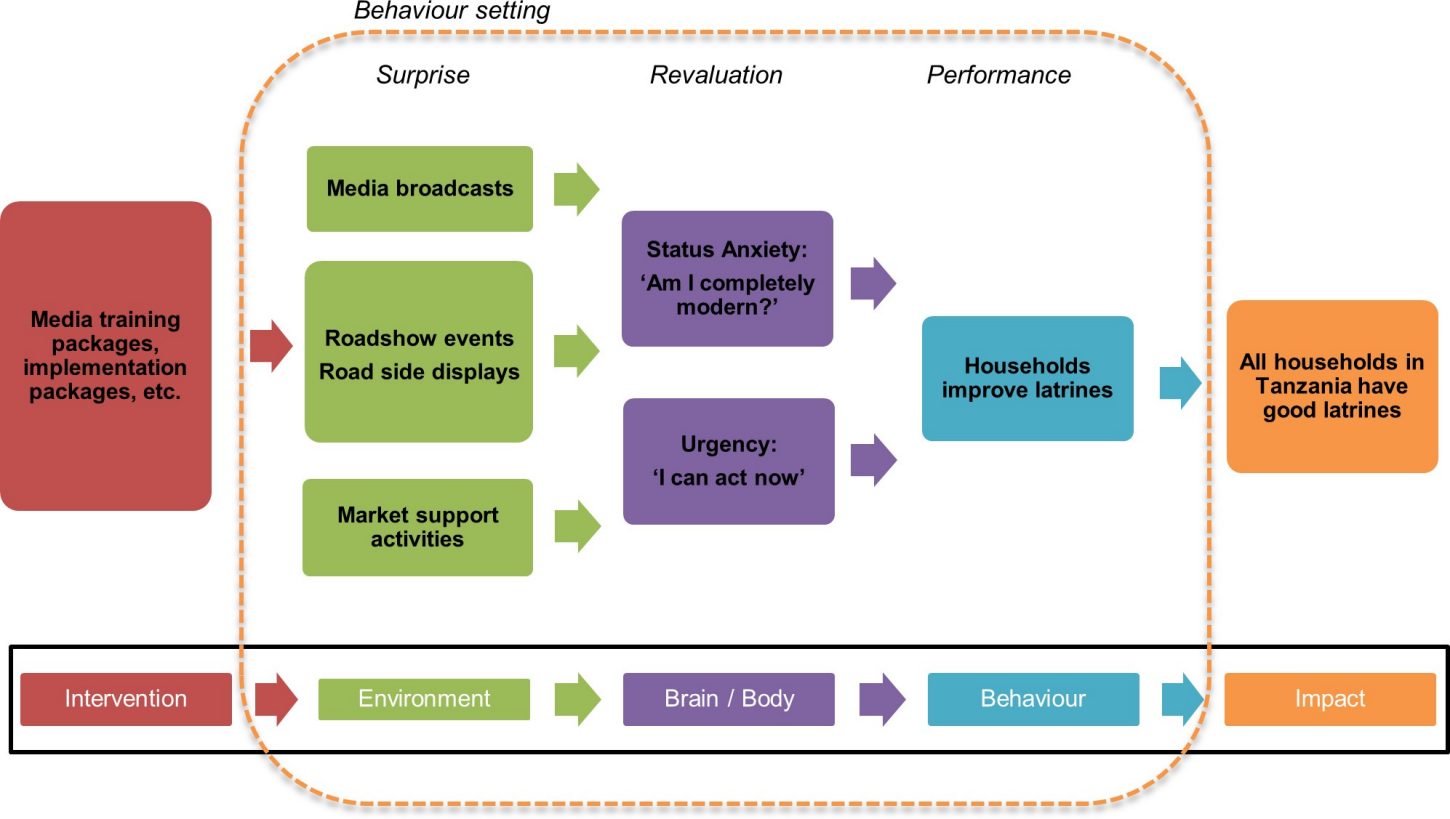
Provisional programmatic Theory of Change.

Fig 2 shows the provisional theory of change for the NSC campaign, informed by this formative research. This provided the basis for the creative brief for the agency who went on to support the design of the campaign.

The FR participants placed a high value on improving their livelihoods over many years through hard work, creative enterprise, enduring setbacks, saving, and developing new skills to improve their surroundings. These results suggest that an effective motivational campaign could be based around an aspiration towards creating a ‘better life’. However, there were two key psychological barriers, which would impede any motive-based campaign: poor quality toilets were not seen as a major problem, and it was normal to take a long time to make such changes, for example, by waiting for an existing toilet to collapse before building a new one. Therefore, toilets had to become problematised and change had to become more urgent.

The desire to create a better, more modern life and to improve social status had led to the majority of households making major improvements to their housing. However, toilets had generally been left out of this ‘home improvement’ process. Toilets were mostly constructed at some distance from homes because of the disgust associated with the smell and sight of these structures. As a result, toilets were ‘out of sight, out of mind’. Toilets were rarely decorated or ‘beautified’, even if elements of an improved latrine type were present. Although disgust has been successfully employed to drive people to move from no sanitation to ‘unimproved’ sanitation elsewhere [34], in these settings disgust was leading to avoidance of the problem.

One potential route to problematising sanitation could be to engage the Status motive, in other words, to generate some ‘status-anxiety’ around the social judgement that could come with owning an unhygienic toilet. Whilst participants were clearly proud of their improved housing, the idea that a poor toilet might reflect badly on the owner was a new idea. Participants, nevertheless, agreed that this might be true. However, the idea that an improved toilet could enhance one’s status was nuanced; the toilet should not be seen to be better than the house, and the owner should not be accused of ‘showing-off’ or poorly prioritising use of their money.

If the value of a *choo bora* could be made more salient, could getting one also become more urgent? Half of participants with poor toilets were considering improving them, but most expected to wait. Participants often cited water scarcity as a real barrier to improving latrines in the short term. Our team believed that this was more of a perceived problem than and environmental one in the areas we visited (although this likely varies across the country). However we acknowledge the need for powerful motivating reasons to overcome the time and inconvenience taken to collect water in this setting. Previous campaigns have employed the Affiliation motive to imply that performing the target behaviours would meet with social approval (and that everyone else was already doing them) [26, 28, 29]. In these settings, changing norms could be a plausible means of making change more urgent. Secondly, the forgotten, but surprising topic of ‘good toilets’ could become part of a village, district and nation-wide conversation. This could be achieved through employing national and local media, by engaging local political champions and by linking these to local events around the topic of toilets. In line with other studies from Tanzania [47, 48] we found traditional gender roles in the households visited. These cultural norms dictated that women were usually responsible for household collection, while men dug latrine pits and made the ultimate economic and spending decisions. Efforts to make toilet improvement more salient and urgent must therefore target men as well as women.

The findings also suggest the need to promote ‘transformational’ toilet improvement through these intervention channels, rather than incremental changes over time such as the ‘Small Doable Actions’ approach [49] or simple ‘nudges’ towards the new behaviour [50]. Improved latrines with a lockable door, a ceramic pan, and cleanable surfaces were desired, but we found little evidence that participants were working towards these improvements in an incremental fashion. We suggest that a ‘home makeover’ type campaign, e.g. as trialled successfully in Nepal [29], might resonate better than the concept of moving gradually up the rungs of the ‘sanitation ladder’ [22] and could be included as one element of the campaign.

Even the wealthier participants showed little urgency to spend hard-earned cash to transform their toilets. For them toilets were expensive, required identifying and contracting a suitable mason as well as researching and committing to a design at the outset. We found that participants often had poor or incomplete information about toilet design and cost. This was demonstrated by the wide range of cost estimates given for a choo bora, the mixed understanding of the components required to build one, and the limited understanding of how to protect their properties and compounds from the effects of poorly constructed or old latrines. This represents a further barrier to performance of the target behaviour (latrine building or improvement). Market support activities are therefore the third strand of the environment modification we believe will instigate the cascade towards behaviour change (Fig 2). There are a number of companies operating in the Tanzania market that offer products and services for toilet improvement (for example the Sato pan made by Lixil (http://www.sato.lixil.com)) that could couple their efforts with the government campaign to offer attractive and affordable options to stimulate behaviour change.

### Methods and limitations

This systematic but flexible approach to FR was a relatively rapid and efficient means of generating actionable insights into the problem of toilets in rural Tanzania. We used engaging tools, meaning that participants were ready to interact with the researchers over extended periods. The flexible nature of the investigation was efficient; for example, once saturation with one tool had been achieved, others could be rotated in, and investigators could maximise time with willing participants, spending less with those who were proving poor informants. The BCD checklist provided clear and comprehensive conceptual guidance to investigators, who were not all experts in behaviour, on the types of behavioural determinants they should pay attention to. The analysis took place in two phases, first in an immediate debriefing workshop, and second in the detailed coding exercise, which is reported here. Much of the richness of the findings was captured in the initial exercise, suggesting that FR for programme design can usefully be conducted in a less academic manner, if concepts are clear, and rigour is maintained through the principled use of the checklist.

In this FR we visited two regions in a country of 31 regions (http://www.nbs.go.tz/). Hence, data do not represent the entire country. The aim of the research was to rapidly assist the creative process, not to collect representative ethnographic information. Our experience in this and in previous formative research projects has been that communities typically show considerable homogeneity with respect to beliefs and practices associated with sanitation and hygiene. However, some studies in Tanzania have found strong religious and cultural trends, for example that sharing of toilets across sexes and generations is unacceptable in some pastoralist communities [51], or that households practicing Christianity or Islam are more likely to report latrine ownership and use than those practicing Traditional African or Pagan religions [52]. These local differences will be important to understand in the roll-out of a national campaign.

## Conclusion

Interventions to improve public health need to be designed based on evidence about behavior in its context. Such evidence usually needs to be collected under pressure of tight deadlines to get programmes up and running, and often with limited budgets. We therefore need approaches to formative research that are rigorous and systematic but rapid and cost-effective. Ideally, such approaches should be easy for non-experts to follow.

Here, an investigation into the determinants of sanitation-related behaviour in rural Tanzania using a variety of interactive and engaging tools provided rapid, actionable insights. Our recommendations guided a creative process that shaped the latrine improvement components of the Government’s current national sanitation campaign. We identified several motives and aspirations that could be employed through mass media channels and community activities to encourage the target behaviour. Firstly, a strong, relevant narrative should associate constructing a better toilet with achieving the common aspiration of a better, more modern life. Second, the campaign should amplify existing feelings of disgust and fear associated with poor quality toilets, and assure the target audience that modest improvements would confer respect and status from the community. The task of the intervention is to change the perception of poor quality latrines as normal and ‘good enough’ by introducing status anxiety linking a good latrine with being a truly modern Tanzanian, and creating a perceived urgency to ‘act now’. Our Theory of Change (based on the Behaviour Centred Design approach) was that a campaign designed in this way would: (1) capture attention in order to bring the problem of toilets into a national and local conversation; (2) cause people to revalue the toilet as a means of achieving the status of being a modern Tanzanian, and (3) make toilet improvement easier through promoting transformational toilet improvements and access to modern toilet products.
